# Dynamic Toolface Estimation for Rotary Steerable Drilling System

**DOI:** 10.3390/s18092944

**Published:** 2018-09-04

**Authors:** Weiliang Wang, Yanfeng Geng, Kai Wang, Jieru Si, Joice de Oliveira Fiaux

**Affiliations:** 1 College of Information and Control Engineering, China University of Petroleum, Qingdao 266580, China; weiliangwang@s.upc.edu.cn (W.W.); s15050648@s.upc.edu.cn (K.W.); s15050670@s.upc.edu.cn (J.S.); 2 School of Petroleum Engineering, China University of Petroleum, Qingdao 266580, China; ls1702021@s.upc.edu.cn

**Keywords:** dynamic Toolface estimation, nonlinear complementary filter, rotary steerable drilling system, accelerometer, gyro

## Abstract

In drilling engineering, Toolface is an angle used to describe bit direction. It is a challenging task to accurately estimate Toolface while drilling because of the downhole harsh conditions, but it is a primary step for the dynamic point-the-bit rotary steerable system (DPRSS). A new dynamic Toolface estimator is present, which fuses measurements from two accelerometers and one gyro. A dual-accelerometer Toolface measuring method is designed to compensate the circumferential acceleration of DPRSS. A nonlinear Complementary Filter (CF) is used to suppress the effect of vibration and axial acceleration. The frequency-domain characteristics of nonlinear CF are analyzed and its natural frequency is determined adaptively based on real time drilling conditions. This new estimator is validated on a DPRSS prototype under typical drilling modes; it is demonstrated with high robustness and follows the references satisfactorily.

## 1. Introduction

Dynamic Point-the-bit Rotary Steerable System (DPRSS) is a novel drilling equipment or technology which builds a wellbore along a predefined trajectory quickly and dramatically reduces the drilling cost [1]. Comparing with the static rotary steerable system, DPRSS reduces friction resistance and reaches larger ultimate horizontal displacements [2]. The benefit of DPRSS mainly comes from its closed-loop control functions, and dynamic Toolface measurement is the primary step. However, the complex downhole drilling conditions, such as circumferential and axial vibration, and more specifically stick-slip, will result in additional measurement noise. There has been little discussion on dynamic Toolface estimation for DPRSS while drilling.

**Figure 1:**
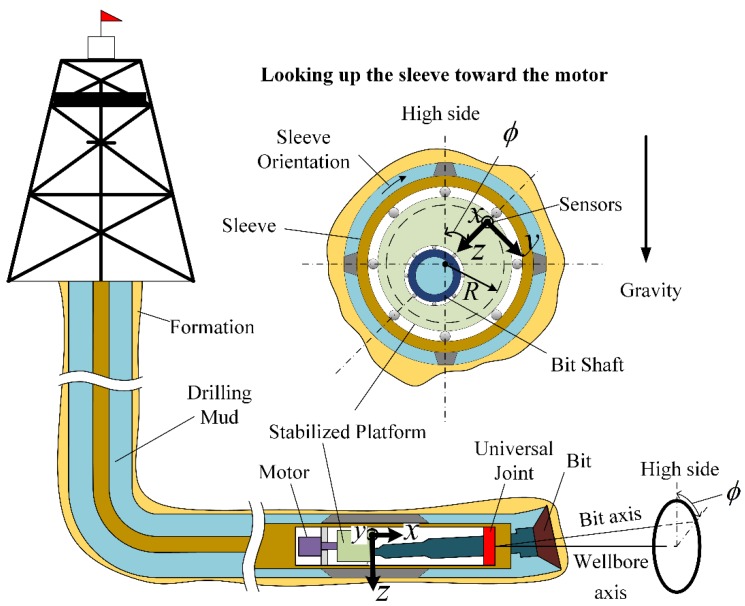
Schematic structure of DPRSS.

A DPRSS structure is shown schematically in Figure 1 [3,4]. The sleeve drilling torque and weight are imposed on the bit through a universal joint. A stabilized platform is assembled inside sleeve and driven by a motor; it has an eccentrically located receptacle which receives one end of the bit shaft. Sensors for Toolface measurement are installed on the surface of the stabilized platform. A left-handed coordinate system is defined in Figure 1, with the *X*-axis pointing to the bit, the *Z*-axis pointing to the center of the stabilized platform, and the *Y*-axis perpendicular to the *XZ* plane. In the *YZ* plane, the top of the borehole is named the high side, and the angle of the *Z*-axis clockwise relative to the high side is named the gravity Toolface, which is denoted by ϕ and is defined between 0 to 360 degrees. Toolface is used to describe bit direction, and it is controlled and adjusted by the stabilized platform.

In practice, there are three different ways to obtain Toolface. The first one is based on the tri-axial accelerometer [5,6], but in the drilling process, besides the kinematic motion acceleration, the drilling shock can be over 200 g [7] and the vibration acceleration is over 5 g [8,9]. Therefore, this method cannot provide an accurate Toolface estimation [5]. The second one is based on angular rate integration. The micro-electro-mechanical system (MEMS) gyro has been used because of its excellent vibration immunity. However, the MEMS gyro has relatively high nonlinearity, random walk and temperature drift due to its inherent drawbacks, and the drift of the MEMS gyro is up to 150°/h [10]. The third one is based on tri-axial magnetometers, such as flux gate. The magnetometers are free from vibration too. There is a theoretical relationship between the measurement results of gravitational accelerometer and magnetometer [11]. However, owing to the motor leakage flux in DPRSS, the operational envelope of magnetometers is limited. Hence, although the accelerometer and the gyro are selected and installed on the DPRSS stabilized platform, it is impossible to obtain an accurate Toolface estimation only through either of the two types of sensors.

There are three problems for the dynamic Toolface estimation. The first one is how to remove the effect of downhole vibration and the acceleration on the gravitational sensor. The second one is how to deal with the gyro drift errors, especially for high temperature applications. The third one is how to develop a high performance data fusion method, through which the strengths of the two types of sensors can be utilized and the gap between them can be bridged.

Based on these findings, a multi-sensor fusion algorithm has been considered. Two classes of multi-sensor fusion technologies are widely used in the measurement field. They are Kalman Filter (KF) and Complementary Filter (CF) [12]. Higgins [13] and Brown [14] illustrated the relationship between them. Higgins points out that CF is actually a steady-state KF. KF-based techniques have been studied in the areas of downhole inclination and azimuth estimation [6,15,16], but most of those methods are complex and impose high computational demand.

Besides the fact that CF is simple and efficient in attitude estimation in contrast with KF [12,17,18], two aspects have also been considered in our selection of CF, namely (1) the gyro measurement is immune to vibration while the accelerometer is sensitive to it; (2) the gyro drift and bias are low frequency signals while the vibration which affects the accelerometer is high frequency signals [19], and the two inertial sensors can complement each other in the frequency domain. Hence, it is possible for the most reliable frequency components of the two sensors measurement to be extracted through CF, and the key factor of CF designing is the selection of natural frequency for different dynamic conditions.

A new Toolface estimation method is proposed, which consists of a dual-accelerometer Toolface measurement and a nonlinear adaptive CF scheme. By analyzing the nonlinear CF frequency characteristics, a constant CF damping ratio was determined and only the CF natural frequency is to be designed. Moreover, the CF natural frequency is determined adaptively by a switch function. Several typical drilling modes were tested on a DPRSS prototype. Toolface estimation results were compared with a motor resolver angular position which is used as a reference in the laboratory. The Toolface estimator showed satisfactory performance in a DPRSS prototype, and future work will focus on its application in actual drilling operations.

This paper is organized as follows: Section 2 provides preliminary knowledge, which includes a short review of CF, Toolface estimation from tri-axial accelerometer and gyroscope. Section 3 shows a comprehensive explanation on the proposed Toolface estimator. Section 4 provides the test results. Section 5 is the conclusion and future work.

## 2. Preliminaries

### 2.1. Toolface Estimation from Tri-Axial Accelerometer

As shown in Figure 1, the tri-axial accelerometer is installed on the surface of the stabilized platform, where the radius is denoted by *R*. The tri-axial accelerometer *X*-axis, *Y*-axis and *Z*-axis measurements are denoted by ${\hat{a}}_{x}$, ${\hat{a}}_{y}$ and ${\hat{a}}_{z}$, respectively. Toolface estimation from the accelerometer is denoted by ${\hat{\phi }}_{a}$ which can be calculated as follows:

(1) $${\hat{\phi }}_{a}=\mathrm{atan}2d({\hat{a}}_{y},{\hat{a}}_{z})$$

When the stabilized platform is rotated with an angular rate p, the ${\hat{a}}_{y}$ and ${\hat{a}}_{z}$ are expressed in Equation (2):

(2) $$\left\{ \begin{array}{l} {\hat{a}}_{y}=g_{yz}\sin\phi -R\dot{p}/2\pi +a_{ey}\\ {\hat{a}}_{z}=g_{yz}\cos\phi -Rp^{2}/4\pi^{2}+a_{ez} \end{array}\right.$$

where $g_{yz}$ is the *YZ* plane gravity component, ϕ is the Toolface real value, and the two terms $R\dot{p}/2\pi$ and $Rp^{2}/4\pi^{2}$ are tangential and centripetal acceleration, which represent motion accelerations, and will make ${\hat{a}}_{y}$ and ${\hat{a}}_{z}$ different from their gravity components. The other two terms $a_{ey}$ and $a_{ez}$ represent additive measurement error arose from the vibration of the stabilized platform and the accelerometer measurement errors. 

Considering the four additional terms, Toolface calculated from Equation (1) is different from its real value, the degree of deviation depending on the magnitude of the terms related to ${\hat{a}}_{y}$ and ${\hat{a}}_{z}$. Additionally, the vibration acceleration is high frequency signals comparing with the variation of Toolface, and can be eliminated by a low-pass filter. However, the centripetal acceleration is a constant bias, which must be removed before using Equation (1).

### 2.2. Toolface Estimation from Gyro

The gyro is also installed on the surface of the stabilized platform, its rate axis parallel with the *X*-axis. The error model used in this paper is [20,21]:

(3) $$\hat{p}=p+b+e_{gyro}$$

where $\hat{p}$ is the gyro measurement, p is the stabilized platform angular rate, and b is the low-frequency time-varying bias; $e_{gyro}$ denotes the additive measurement noise which is assumed to be white noise. The Toolface is obtained by:

(4) $${\hat{\phi }}_{g}=\int \hat{p}dt+\phi_{ini}==\phi +\phi_{g\_bias}+\phi_{ini}$$

where, ${\hat{\phi }}_{g}$ is the gyro Toolface estimation, $\phi_{ini}$ is the Toolface initial value, and $\phi_{g\_bias}$ denotes the gyro integration error from bias and measurement noise. It is clear that the accuracy of ${\hat{\phi }}_{g}$ is affected by $\phi_{g\_bias}$ and $\phi_{ini}$.

### 2.3. Complementary Filter

CF is a distortionless filter or all pass filter [21] which is designed with a high pass filter (HPF) and a low pass filter (LPF). For Toolface estimation, CF combines high frequency components from ${\hat{\phi }}_{g}$ and the low frequency components from ${\hat{\phi }}_{a}$. As such, true Toolface is obtained while noise is filtered as desired.

**Figure 2:**
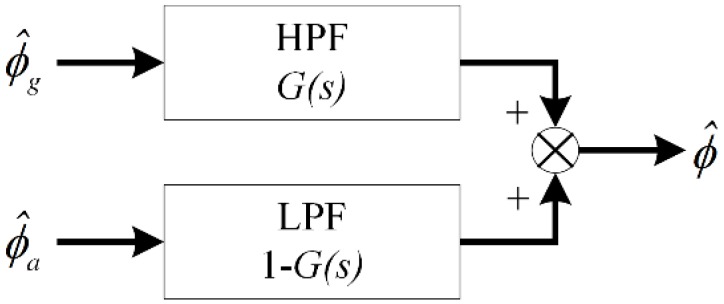
Basic Structure of CF.

The basic structure of CF is shown in Figure 2. Let G(s) be the HPF transfer function and 1−G(s) is the complementary LPF transfer function; the function of CF is given as:

(5) $$\hat{\phi }={\hat{\phi }}_{g}G(s)+{\hat{\phi }}_{a}(1-G(s))$$

For the noiseless measurement, the G(s) can be defined as a constant, i.e., G(s)=α, α∈[0,1], α being the termed weighting factor. This type of CF is named linear CF [22] or first order CF [12], but it does not act as HPF or LPF.

**Figure 3:**
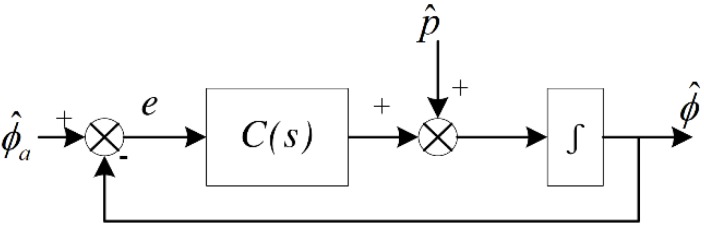
Block diagram of basic CF.

In practice, CF is reconfigured to a control system scheme which is shown in Figure 3, where C(s) has the same meaning as a controller. In this case, CF is easy to implement and the classical controller design techniques can be used in filter design. 

If C(s) is a proportional controller, i.e., $C(s)=K_{p}$, the function of CF is given by:

(6) $$\hat{\phi }=\frac{\hat{p}}{s}\frac{s}{s+K_{p}}+{\hat{\phi }}_{a}\frac{K_{p}}{s+K_{p}}$$

where *s* is the Laplace variable and the gain $K_{p}$ is cut-off frequency (rad/s). The error equation of Equation (6) is:

(7) $$\delta \hat{\phi }=\frac{\delta \hat{p}}{s}\frac{s}{s+K_{p}}+\delta {\hat{\phi }}_{a}\frac{K_{p}}{s+K_{p}}$$

where $\delta \hat{\phi }$ is the final Toolface estimation error, $\delta \hat{p}$ is the gyro measurement error, and $\delta {\hat{\phi }}_{a}$ is the accelerometer measurement error. Applying the final value theorem to Equation (7),

(8) $$\underset{t\to \infty }{\lim}\delta \hat{\phi }=\frac{\delta \hat{p}}{K_{p}}+\delta {\hat{\phi }}_{a}$$

In Equation (8), the final value of $\delta \hat{\phi }$ is not zero. In order to improve the performance of CF, a proportional-integral (PI) controller is introduced:

(9) $$C(s)=K_{p}+\frac{K_{i}}{s}$$

Then, the function of CF is given by:

(10) $$\hat{\phi }=\frac{\hat{p}}{s}G_{HP}(s)+{\hat{\phi }}_{a}G_{LP}(s)$$

where,

(11) $$\left\{ \begin{array}{l} G_{HP}(s)=\frac{s^{2}}{s^{2}+K_{p}s+K_{i}}\\ G_{LP}(s)=\frac{K_{p}s+K_{i}}{s^{2}+K_{p}s+K_{i}} \end{array}\right.$$

The $G_{HP}(s)$ is an HPF transfer function and $G_{LP}(s)$ is the complementary LPF transfer function. Applying the final value theorem to Equation (10):

(12) $$\underset{t\to \infty }{\lim}\delta \hat{\phi }=\delta {\hat{\phi }}_{a}$$

The CF shown in Equation (10) is named the nonlinear CF, its estimation error converges to the accelerometer measurement error, and the gyro measurement error does not exist in Equation (12). 

## 3. Toolface Estimation Method

### 3.1. Dual-Accelerometer Toolface Measurement

As previously mentioned in Section 2, the low frequency component of ${\hat{\phi }}_{a}$ and the high frequency component of ${\hat{\phi }}_{g}$ are fused by nonlinear CF, however, the motion acceleration of the stabilized platform has effects on low frequency component of ${\hat{\phi }}_{a}$. To remove this effect, a dual-accelerometer Toolface measurement method is proposed.

**Figure 4:**
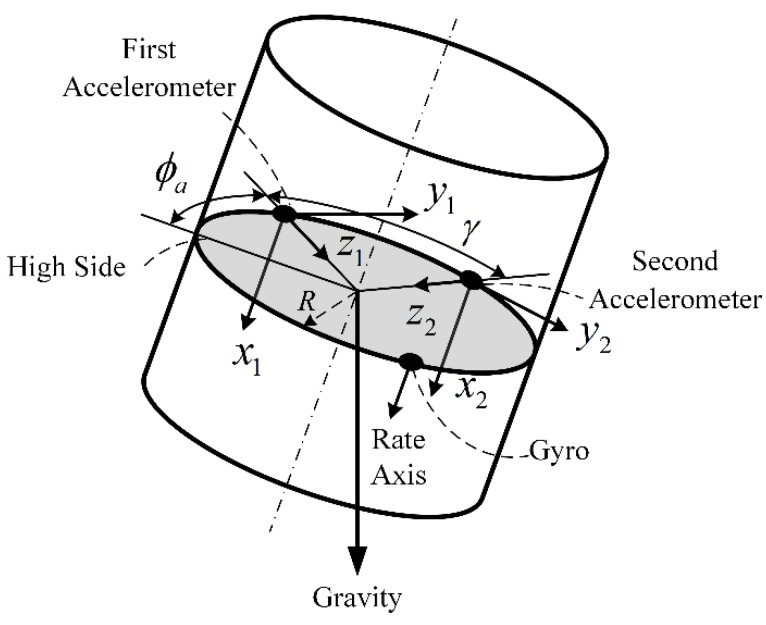
Schematic diagram of dual-accelerometer Toolface calculation.

As shown in Figure 4, two accelerometers are installed on the surface of stabilized platform, the angle between two accelerometers is denoted by γ, the *Y*-axis and *Z*-axis measurements are as follows:

(13) $$\left\{ \begin{array}{l} {\hat{a}}_{y_{1}}=g_{yz}\sin\phi_{a}-R\dot{p}/2\pi +a_{ey_{1}}\\ {\hat{a}}_{z_{1}}=g_{yz}\cos\phi_{a}-Rp^{2}/4\pi^{2}+a_{ez_{1}}\\ {\hat{a}}_{y_{2}}=g_{yz}\sin(\phi_{a}+\gamma )-R\dot{p}/2\pi +a_{ey_{2}}\\ {\hat{a}}_{z_{2}}=g_{yz}\cos(\phi_{a}+\gamma )-Rp^{2}/4\pi^{2}+a_{ez_{2}} \end{array}\right.$$

where the subscript 1 and 2 represent the first and the second accelerometer, respectively, while the other terms are the same as Equation (2). When the two accelerometers are oppositely placed, i.e., $\gamma =180^{\circ }$, the $a_{ey_{1}}$ is equal to $-a_{ey_{2}}$ and $a_{ez_{1}}$ is equal to $-a_{ez_{2}}$, we can obtain:

(14) $$\left\{ \begin{array}{l} {\hat{a}}_{xf}=({\hat{a}}_{x_{1}}+{\hat{a}}_{x_{2}})/2\\ {\hat{a}}_{yf}={\hat{a}}_{y_{1}}-{\hat{a}}_{y_{2}}=2g_{yz}\sin\phi_{a}+a_{ey_{1}}-a_{ey_{2}}=2g_{yz}\sin\phi_{a}+2a_{ey_{1}}\\ {\hat{a}}_{zf}={\hat{a}}_{z_{1}}-{\hat{a}}_{z_{2}}=2g_{yz}\cos\phi_{a}+a_{ez_{1}}-a_{ez_{2}}=2g_{yz}\cos\phi_{a}+2a_{ez_{1}} \end{array}\right.$$

where ${\hat{a}}_{yf}$ and ${\hat{a}}_{zf}$ are the processed measurements. The dual-accelerometer measurement method cannot be used to correct the *X*-axis measurement, which is denoted by ${\hat{a}}_{xf}$, in the case of estimating downhole total acceleration, the average of ${\hat{a}}_{x_{1}}$ and ${\hat{a}}_{x_{2}}$ is used as ${\hat{a}}_{xf}$. In Equation (14), the terms $R\dot{p}/2\pi$ and $Rp^{2}/4\pi^{2}$, which represent the motion acceleration, are removed, while the *YZ* plane gravity component $g_{yz}$ and the vibration acceleration terms $a_{ey}$ and $a_{ez}$ are magnified with same factor, so the Dual-accelerometer Toolface measurement ${\hat{\phi }}_{af}$ can be calculated as the following:

(15) $${\hat{\phi }}_{af}=\mathrm{atan}2d({\hat{a}}_{yf},{\hat{a}}_{zf})$$

It is clear that Toolface has a singularity at 360° [23]. In practice, ${\hat{\phi }}_{a}$ and $\hat{\phi }$ occur across 360° at a different time due to sensor measurement delay, thus, when an undesired large error $e={\hat{\phi }}_{a}-\hat{\phi }$ arises, it reduces CF performance. To realize a full range accurate Toolface estimation, a preprocessing method is used which limits errors between −180° and 180°.

(16) $$e=\{\begin{array}{ll} {\hat{\phi }}_{a}-\hat{\phi }+360; & if\;{\hat{\phi }}_{a}-\hat{\phi }<-180\\ {\hat{\phi }}_{a}-\hat{\phi }-360; & if\;{\hat{\phi }}_{a}-\hat{\phi }\geq 180\\ {\hat{\phi }}_{a}-\hat{\phi }; & else \end{array}$$

### 3.2. Toolface Adaptive Nonlinear CF Scheme

The CF performance is determined by $K_{p}$ and $K_{i}$. Based on Equation (11) and the classical frequency design method [18], the mathematical relationship for $K_{p}$ and $K_{i}$ are given as Equation (17):

(17) $$\left\{ \begin{array}{l} K_{p}=2\zeta \omega_{n}\\ K_{i}=\omega_{n}{}^{2} \end{array}\right.$$

where $\omega_{n}$ is the natural frequency, rad/s; ζ is the damping ratio. In order to simplify the CF parameter design, the damping ratio is selected as a constant, and the natural frequency is to be tuned for satisfactory CF performance.

#### 3.2.1. Frequency-Domain Characteristics of Nonlinear CF

The $G_{HP}(s)$ and $G_{LP}(s)$ magnitude curves intersection is denoted by $\left(\omega_{t},M_{t}\right)$, $\omega_{t}$ is the intersection frequency and $M_{t}$ is the intersection magnitude. Let

(18) $$\left|G_{HP}(j\omega_{t})\right|=\left|G_{LP}(j\omega_{t})\right|$$

One obtains:

(19) $$\left\{ \begin{array}{l} \omega_{t}=\omega_{n}\sqrt{2\zeta^{2}+\sqrt{4\zeta^{4}+1}}\\ M_{t}=\frac{2\zeta^{2}+\sqrt{4\zeta^{4}+1}}{\sqrt{{\left(1-(2\zeta^{2}+\sqrt{4\zeta^{4}+1})\right)}^{2}+4\zeta^{2}(2\zeta^{2}+\sqrt{4\zeta^{4}+1})}} \end{array}\right.$$

The resonance points of $G_{HP}(s)$ and $G_{LP}(s)$ are denoted by $\left(\omega_{r\_HP},\;M_{r\_HP}\right)$ and $\left(\omega_{r\_LP},\;M_{r\_LP}\right)$ respectively. Let

(20) $$\left\{ \begin{array}{l} \frac{d\left|G_{HP}(j\omega_{r\_HP})\right|}{d\omega_{r\_HP}}=0\\ \frac{d\left|G_{LP}(j\omega_{r\_LP})\right|}{d\omega_{r\_LP}}=0 \end{array}\right.$$

Then one obtains,

(21) $$\left\{ \begin{array}{l} \omega_{r\_HP}=\omega_{n}\frac{1}{\sqrt{1-2\zeta^{2}}}\\ M_{r\_HP}=\frac{1}{2\zeta \sqrt{1-\zeta^{2}}}\\ \omega_{r\_LP}=\frac{\omega_{n}\sqrt{\sqrt{8\zeta^{2}+1}-1}}{2\zeta }\\ M_{r\_LP}=\frac{4\xi^{2}{\left(8\xi^{2}+1\right)}^{\frac{1}{4}}}{\sqrt{16\xi^{2}+2+(16\xi^{4}-8\xi^{2}-2)\sqrt{8\xi^{2}+1}}} \end{array}\right.$$

From Equations (19) and (21), it can be seen that the magnitudes of the intersection point and resonance point are determined by ζ, while the frequencies of the two points are related to $\omega_{n}$ and ζ.

**Figure 5:**
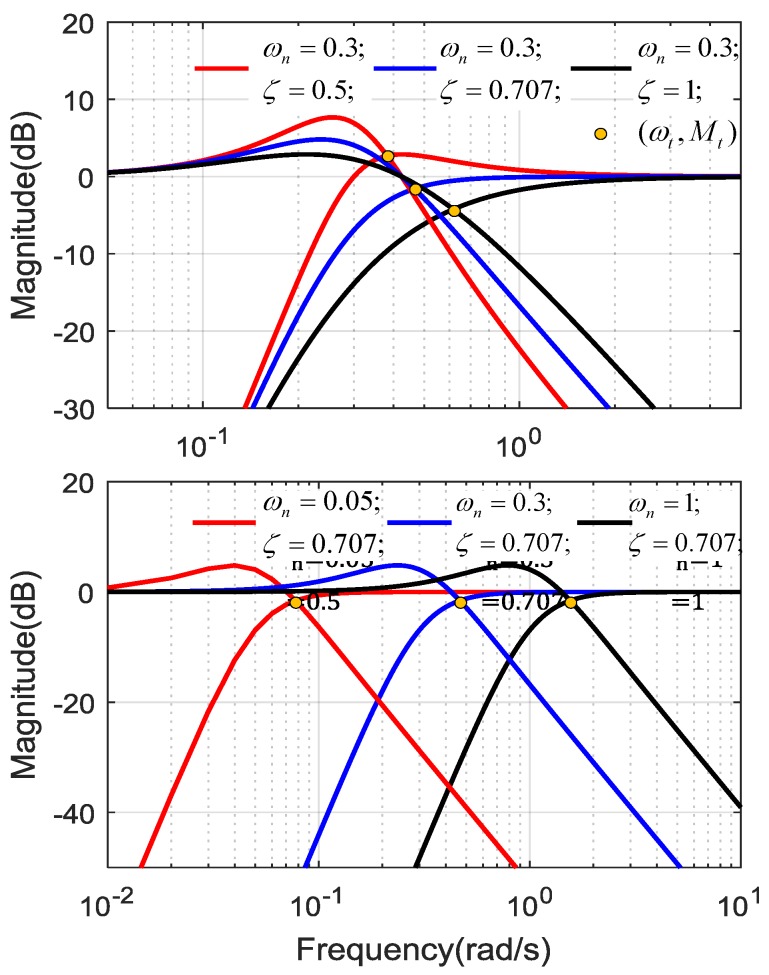
Magnitude diagram of complementary filter.

The nonlinear CF magnitude curves with different ζ and $\omega_{n}$ are shown in Figure 5. In the first graph, $\omega_{n}$ is fixed, the filters’ magnitude curves are different from each other, and the magnitude of LPF and HPF depended on the value of ζ. All of the intersection frequencies are larger than $\omega_{n}$, and increase with ζ. In the second graph, ζ is fixed, the shape of magnitude curves is the same, but intersection frequencies are still larger than corresponding $\omega_{n}$. CF noise suppressing performance is closely related with the magnitude curve, especially the curve shape in the vicinity of resonance frequency, which can be designed by selecting proper ζ, and the trade-off between HPF and LPF can be realized by selecting $\omega_{n}$.

In literature, ζ is designed experimentally [18,24,25]. From Equations (19) and (21), it can be seen that when ζ≥0.707, the HPF do not have resonance due to $\omega_{r\_HP}$ only having imaginary solutions, and $\underset{\zeta \to \infty }{\lim}M_{r\_LP}=0\;\mathrm{dB}$, which reveals that the LPF resonance cannot be removed.

**Figure 6:**
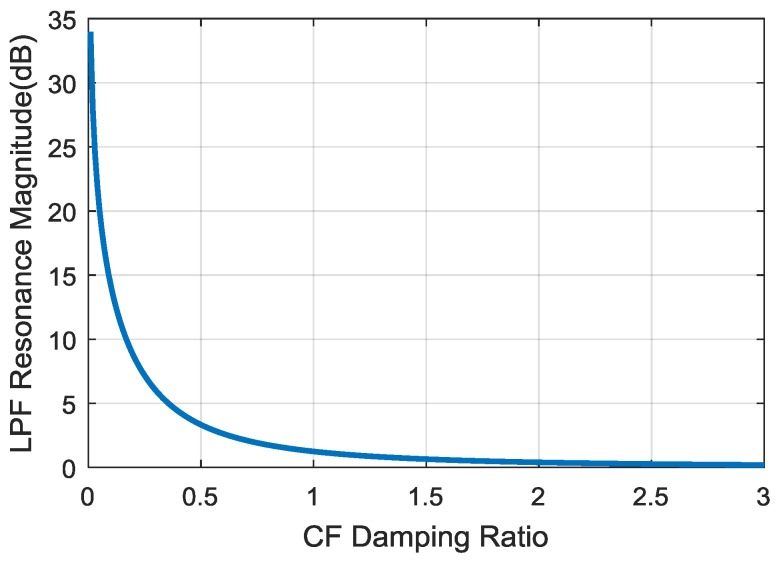
Relationship between LPF Resonance Magnitude and CF Damping Ratio.

Figure 6 presents the relationship between $M_{r\_LP}$ and ζ, the $M_{r\_LP}$ is plotted in dB units. In this graph, $M_{r\_LP}$ and ζ have a negative nonlinear correlation, while small $M_{r\_LP}$ can be obtained by increasing ζ. However in Figure 5, large ζ leads to a small roll-off rate, and the design of CF seeks to make the roll-off as narrow as possible. In this case, the CF performance will be close to its ideal design.

#### 3.2.2. Adaptive Nonlinear CF Scheme

In order to make a narrow roll-rate and remove HPF resonance, ζ is fixed to 0.707, thus, $M_{r\_LP}$ is fixed to 2.1 dB, then, the CF is only characterized by $\omega_{n}$. A larger $\omega_{n}$ means a more high frequency component of sensors will be included in Toolface estimation of CF output, hence, a better dynamic response is obtained, while a smaller $\omega_{n}$ means the high frequency components of sensors will be filtered, and Toolface estimation will steadily follow the major trends of real Toolface.

Various types of CF parameter designing method have been proposed. A fixed weight factor is implemented in linear CF [12], but the fixed gains cannot converge properly under complex circumstances. While neglecting any motion accelerations, the error *e* which is shown in Figure 3 is used as a cost-function, then intelligence stochastic searching algorithm [22] and the least square error method [26] are introduced to obtain optimal $K_{p}$ and $K_{i}$. Gain-scheduled CF is introduced [18], the gains are selected from three predetermined values and switched discontinuously. To improve CF performance, the gain-scheduling rules must contain more scenarios and be fine-tuned. The fuzzy logic based CF gain switch logic is commonly adopted [24,25,27,28] to continuously switch gains based on error levels, but it leads to increased computational time, as the fuzzy logic parameters tuning is a complex task.

The dynamic intensity was usually described by absolute acceleration magnitude, by which the $\omega_{n}$ switch function is designed [18,25,27], but in the drilling process, the additional acceleration is strong and always exists, the absolute acceleration magnitude are most likely to lead to incorrect CF gain switches.

Due to the acceleration magnitude being positively correlated with vibration severity, the variance of acceleration magnitude is used as the vibration factor in $\omega_{n}$ calculation:

(22) $$\left\{ \begin{array}{l} A=Var(\sqrt{\left({\hat{a}}_{xf}{}^{2}+{\hat{a}}_{yf}{}^{2}+{\hat{a}}_{zf}{}^{2}\right)})\\ P=Var(\left|\hat{p}\right|) \end{array}\right.$$

where A is the variance of the acceleration magnitude. Similarly, the variance of rotation rate denoted by P is another factor in $\omega_{n}$ calculation. In practice, the A and P are calculated by rolling variance, they are updated during each sampling period. 

**Figure 7:**
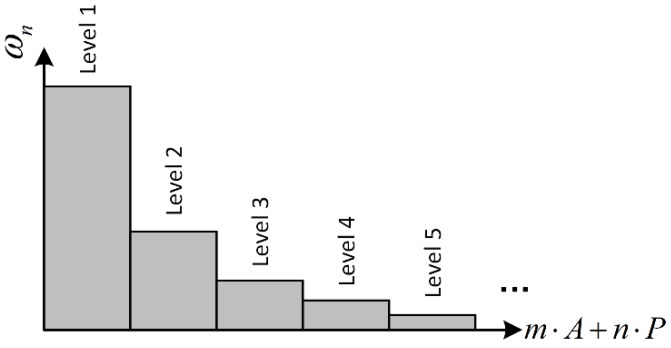
CF gain-scheduling rules.

The dynamic intensity is described by m⋅A+n⋅P, where the *m* and *n* are two weight factors of vibration and angular rate. Based on the rules designed by Hong [18], the general $\omega_{n}$ gain-schedule rule is shown in Figure 7, the dynamic intensity is separated into several levels, and the best $\omega_{n}$ for each levels is determined by experimental data and off-line optimization [29].

In Figure 7, as the dynamic intensity increases, the difference of $\omega_{n}$ between two dynamic intensity levels becomes small. Obviously, the exponential function is a good selection to fit or approximate the relationship between $\omega_{n}$ and dynamic intensity, so the $\omega_{n}$ switch function is designed as follows:

(23) $$\omega_{n}=\Omega \cdot e^{-m\cdot A-n\cdot P}$$

where Ω is the maximum of $\omega_{n}$, rad/s. It should be noted that the switch function is not unique, but the Equation (23) is simple and shows satisfactory performance during our experimental tests. The CF natural frequency can be determined based on the dynamic intensity adaptively and continuously.

There are two methods to design Ω: (1) Ω can be set to be equal to $\omega_{n}$ when vibration and motion acceleration are relatively low; (2) ${\hat{\phi }}_{af}$ low frequency information can be obtained by Fast Fourier Transformation analysis, then we can define the passband of ${\hat{\phi }}_{af}$, and Ω can be slightly larger than the passband. According to Zhou and Liu [30,31], when the low-pass filter cut-off frequency is less than 0.2 Hz, the vibration acceleration affections can be eliminated, after analyzing several ${\hat{\phi }}_{af}$ frequency magnitude curves, we choose Ω to be 0.3 rad/s (0.05 Hz).

The two weight factors *m* and *n* define how fast does $\omega_{n}$ change regarding the vibration and angular rate. Due to most of the motion acceleration effect being complemented by the dual-accelerometer measurement method, vibration is the key factor to be considered in the CF scheme. A simple way to tune *m* and *n* is: Setting *n* to 0 at the beginning of parameter tuning, then *m* can be tuned, after that, increasing *n* slowly until Toolface estimation satisfies the accuracy requirements. In this way, *m* and *n* might not be optimal, but it is easy to carry out the tuning in practice.

**Figure 8:**
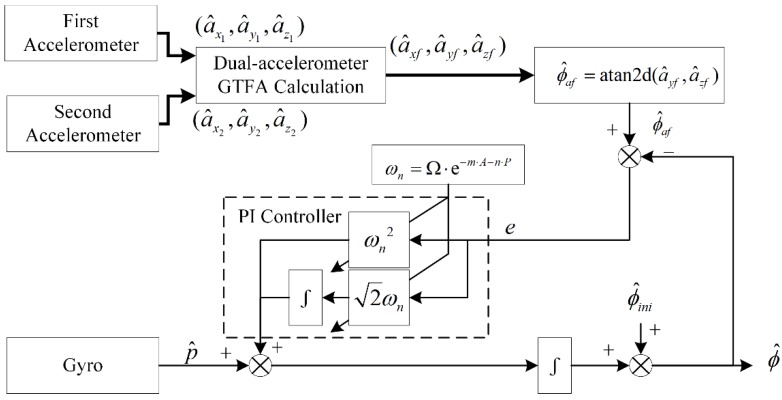
Block diagram for proposed adaptive nonlinear CF scheme.

The proposed dynamic Toolface estimation nonlinear CF scheme is shown in Figure 8. The figure denotes estimated initial Toolface as ${\hat{\phi }}_{ini}$, then $\hat{\phi }$ can be calculated:

(24) $$\hat{\phi }=\sum ((\omega_{n}{}^{2}e+\sqrt{2}\omega_{n}\sum e)+\hat{p})+{\hat{\phi }}_{ini}$$

Dynamic Toolface is obtained in the following steps:

**Step** **1:** Initialization: Acquire data from dual-accelerometer in steady state, calculate ${\hat{\phi }}_{af}$ by Equation (15), let $\hat{\phi }={\hat{\phi }}_{af}$ and ${\hat{\phi }}_{ini}={\hat{\phi }}_{af}$;
**Step** **2:** Start estimation: Calculate ${\hat{\phi }}_{af}$ and e by Equation (15) and Equation (16), respectively;
**Step** **3:** Calculate A and P by Equation (22), then obtain $\omega_{n}$ from Equation (23);
**Step** **4:** Calculate $\hat{\phi }$ by Equation (24), GOTO step 2 for next instant.

## 4. Experiments and Results

### 4.1. DPRSS Prototype for Experiments

**Figure 9:**
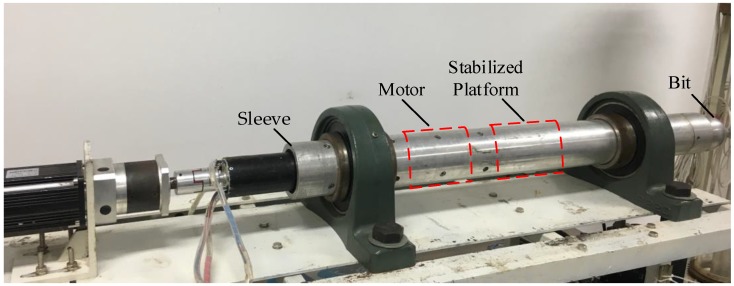
DPRSS Prototype.

As shown in Figure 9, a DPRSS prototype is developed. The sleeve is horizontally placed on a fixed stand. The stabilized platform, which is driven by motor, is assembled inside the sleeve. Two MMA8451 accelerometers and an HTG-1200 gyro are mounted on a stabilized platform in the manner shown in Figure 4, where *R* = 0.025 m, and the sensors specifications are shown in Table 1. A 16-bit AD converter ADS8320 is used to acquire the output of gyro. An MC9S12XS128 microprocessor is attached to the accelerometers and AD converter via serial communication. A resolver which can measure motor rotor position and angular rate is installed at the end of the motor, a 14-bit R/D converter is used to convert the resolver analog output to a digital signal. A TMS320F28335 DSP is attached to the R/D converter via SPI communication, and the DSP is also used to control the motor velocity and rotor position. All data were acquired via USB-CAN analyzer with a 200 Hz sample rate and processed by Matlab software. During the experiment, the sleeve was non-rotational, and the motor rotor position denoted by ${\hat{\phi }}_{ref}$ was considered to be the Toolface reference, the root mean square error (RMSE) of estimation results was selected as an index of CF performance.

### 4.2. Dual-Accelerometer Toolface Measurement Test

**Figure 10:**
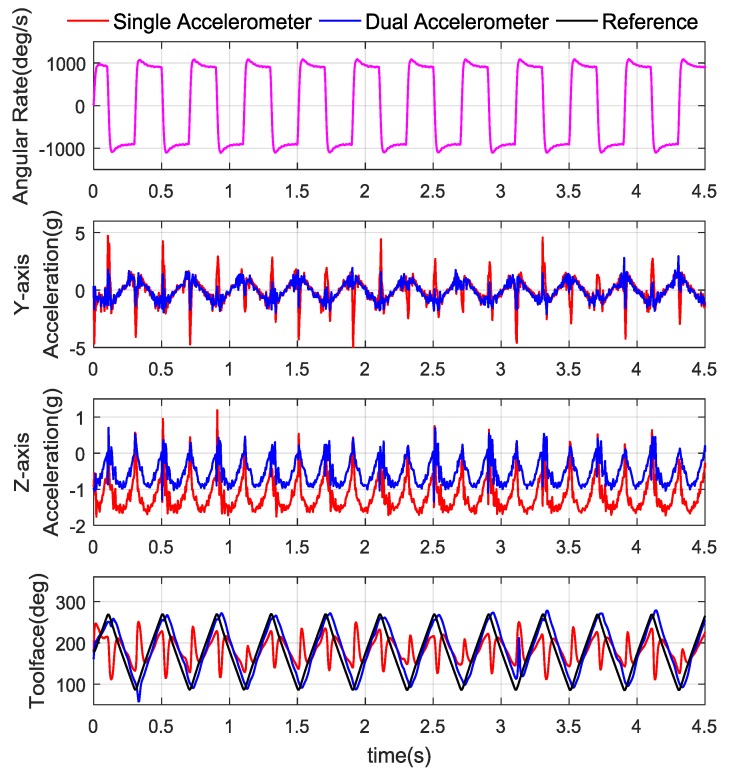
Stabilized platform angular rate, raw accelerometer data, and Toolface comparison.

The references, raw accelerometer output and dual-Toolface measurement results are shown in Figure 10. The stabilized platform angular rate was switched based on a square wave.

The stabilized platform setting angular rate was ±900°/s, it was a straight forward process to calculate that the maximum tangential acceleration and centripetal acceleration were 2.7 g and 0.638 g, respectively. In Figure 10, the *Y*-axis maximum difference between the single accelerometer and the dual-accelerometer measurement is 2.8 g and the *Z*-axis maximum value is 0.628 g, which is in accordance with theoretical analysis, while the motion acceleration is suppressed. The RMSE of the single accelerometer and dual accelerometer are 52.7° and 24.9°, respectively. It should be noted that though a nearly motion-free measurement has been obtained, the vibration effect still needs to be considered.

### 4.3. Dynamic Toolface Estimator Performance Test

The DPRSS prototype is placed horizontally and firmly fastened to a vibration platform. The vibration platform generates vertical vibration with variance frequency of between 0 and 50 Hz. In the test, A and P which are shown in Equation (22) are calculated by rolling variance, a small relative rolling window size shows more details while a relative large rolling window size shows more major trends, after trying various length of rolling windows, the window size is set to 10. In future research, the length of rolling windows should be adjusted according to the frequency properties of A and P.

The stick-slip phenomenon commonly exists in the drilling process, it is a mode of torsional vibrations of a drilling assembly, and its vibration baseband is less than 0.5 Hz [8,9]. Toolface variation rang is less than 20° in DPRSS porotype, a sine curve with 20° magnitude and 0.5 Hz frequency was designed to simulate the stick-slip vibration.

#### 4.3.1. Stick-Slip Dynamic Toolface Estimator Test and Parameters Tuning

The tests were designed consisting of steady state and stick-slip state, while both non-vibration and vibration conditions were considered. There are four successive states: The first one is non-vibration and non-stick-slip, the second one is non-vibration and stick-slip, the following one is vibration and non-stick-slip, the last one is vibration and stick-slip.

**Figure 11:**
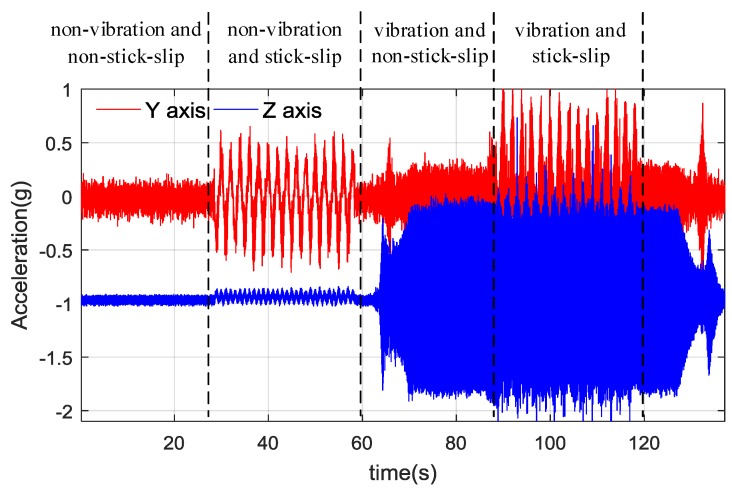
*Y* and *Z* axis measurements.

The dual-accelerometer *Y*-axis and *Z*-axis measurements are depicted in Figure 11. During the experiment, Ω was 0.3 rad/s, *m* was selected between 0 to 50 with equal intervals, *n* was selected in the same way, the raw sensor data in Figure 11 was used for testing. By carrying out rigorous sets of experiment with different parameters, the effect of *m* and *n* values on Toolface estimation performance was studied.

**Figure 12:**
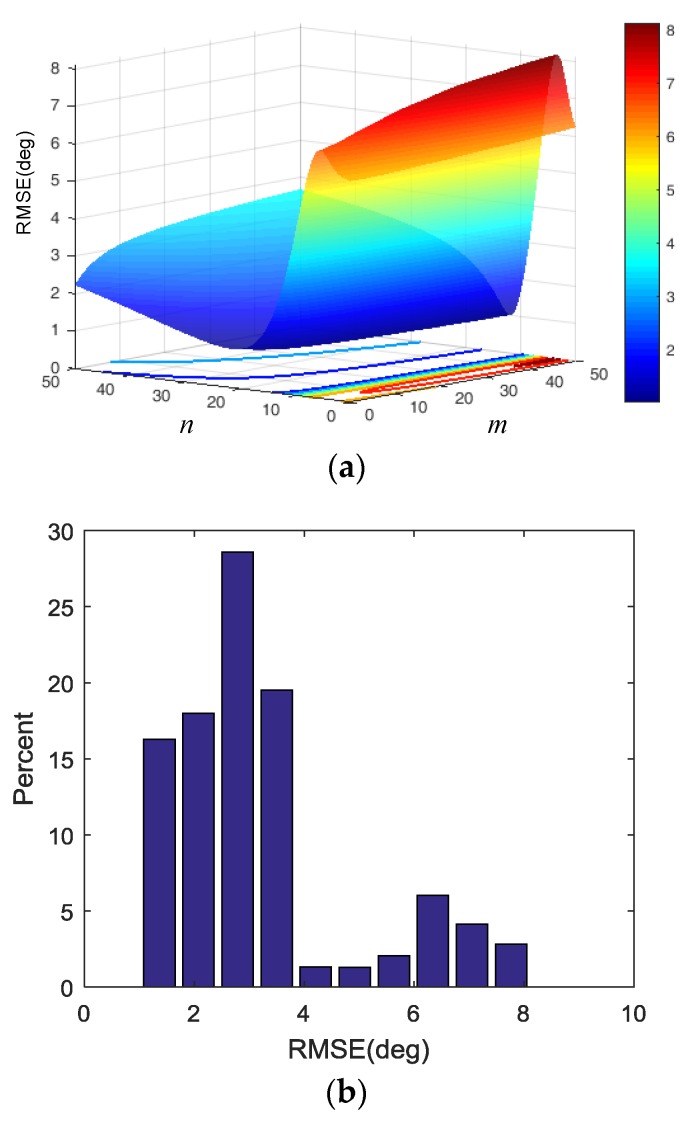
Parameter tuning results. (**a**) RMSE value with different *m* and *n*. (**b**) RMSE distribution histogram.

In Figure 12, RMSE of dynamic Toolface estimator varies with the selection of different *m* and *n*, but they are all less than 8.2°, whereas over 82% of the RMSE values are less than 4°. When *m* = 12.12 and *n* = 45.96, the least RMSE is obtained. In fact, due to *m* and *n* only changing the increase or decrease rate of $\omega_{n}$ for specific vibration and motion acceleration, these parameters do not have a significant impact on CF performance, as the adaptive CF scheme provides robustness against different *m* and *n* values.

**Figure 13:**
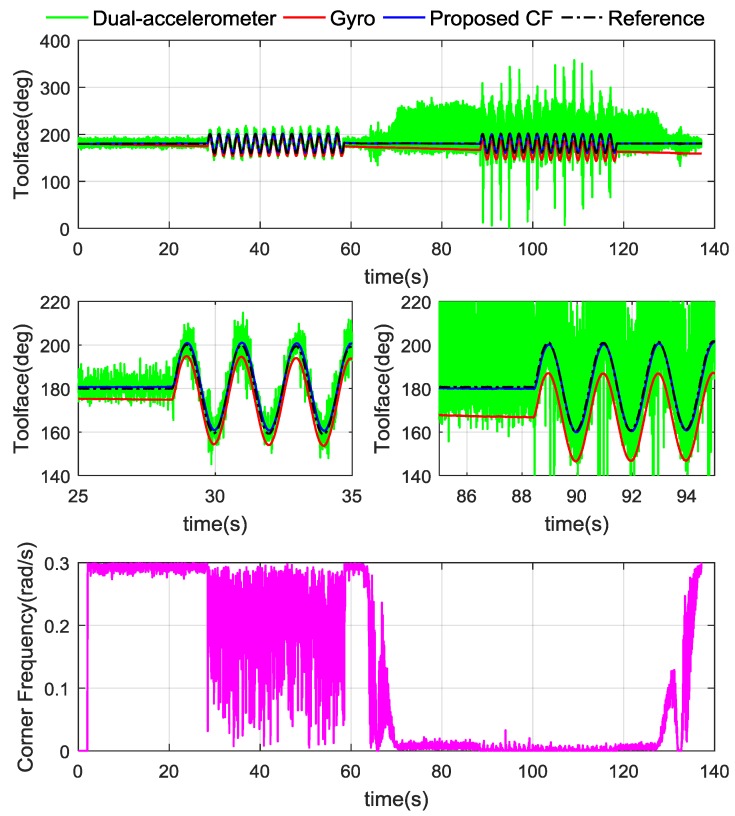
Toolface estimation results and CF natural frequency in stick-slip test.

Let Ω=0.3, ξ=0.707, m=12.12 and n=45.96, the Toolface estimation results are shown in Figure 13. To initialize CF, $\omega_{n}$ of the first 2 s was 0 rad/s. It can be seen that ${\hat{\phi }}_{af}$ follows the reference steadily, but ${\hat{\phi }}_{g}$ has gradual instability of integration drifting. The CF natural frequency $\omega_{n}$ is switched as desired, more ${\hat{\phi }}_{af}$ is used in the low acceleration state and more ${\hat{\phi }}_{g}$ is used under high vibrations. By fusing ${\hat{\phi }}_{af}$ and ${\hat{\phi }}_{g}$ with the proposed CF scheme, $\hat{\phi }$ trends to follow ${\hat{\phi }}_{ref}$ satisfactorily. According to Table 2, the RMSE of proposed CF scheme is 1.0069°, which is improved major improvement over the other two methods.

#### 4.3.2. Multi Processes Dynamic Toolface Estimator Test

**Figure 14:**
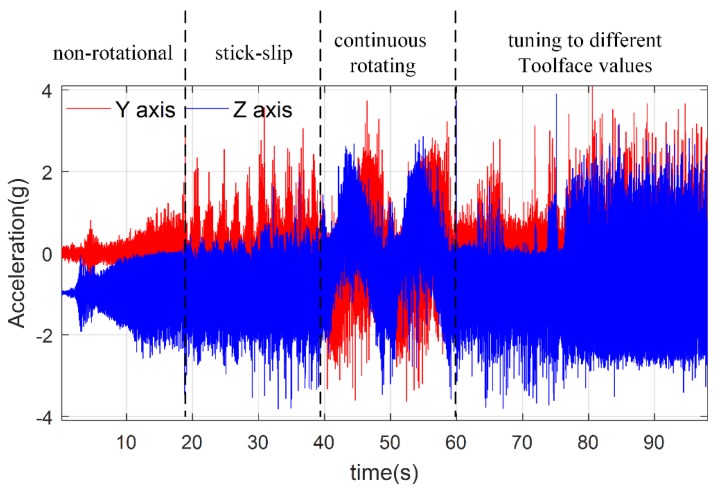
The *Y*-axis and *Z*-axis acceleration measurements.

Four typical processes were designed to test the performance of dynamic Toolface estimator. Firstly, steady state, where the stabilized platform was non-rotational. Next is stick-slip. Thirdly, the stabilized platform rotated continuously, which was used to test full rang estimator performance. Finally, the stabilized platform was turned to different Toolface values, which is named the Toolface orientation drilling process. Dual-accelerometer measurements are shown in Figure 14, vibration effect and stick-slip motion effect can be observed.

**Figure 15:**
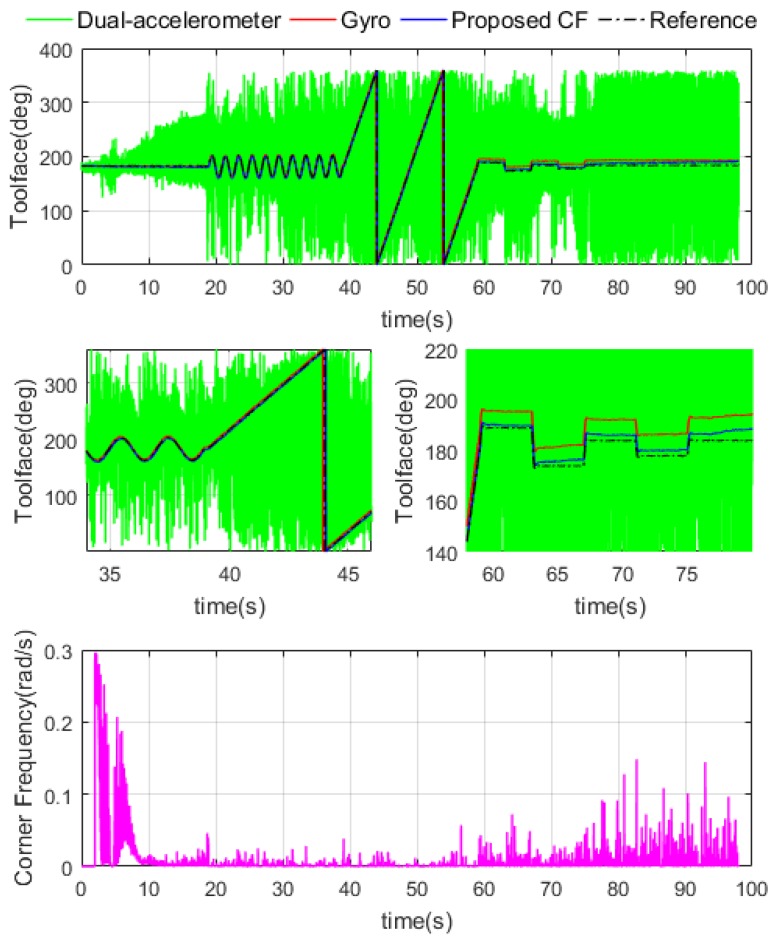
Toolface estimation results and CF natural frequency in multi processes test.

The same parameters as Section 4.3.1 are used in this test, the performance of a well-designed estimator under different dynamic conditions was shown in Figure 15. Toolface from dual-accelerometer measurement, gyro and proposed estimator are plotted together. It is clear that dual-accelerometer measurement is affected by vibration, the gyro estimation is accurate at the beginning but shows gradual instability of integration drifting, and the proposed estimator shows promising performance in full range. The CF natural frequency values are shown in the fourth graph of Figure 15. Due to high dynamic intensity, the CF natural frequency remains relatively small, and more high frequency gyro components are used in CF results.

The RMSE of different Toolface results are demonstrated in Table 3, the robustness of the proposed CF under multi-drilling processes with vibration was shown. The data leads us to the conclusion that a well-designed Toolface estimator can offer a satisfactory performance. The proposed method could be usefully employed in actual DPRSS dynamic Toolface estimation.

## 5. Conclusions

The dynamic Toolface estimation is a key aspect of DPRSS. The complex drilling vibration and motion acceleration seriously affect the accuracy of Toolface measurement. A new dynamic Toolface estimator is proposed which is based on the measurements from two accelerometers and one gyro. A dual-accelerometer Toolface measurement method is used for complementing motion acceleration efficiently. An adaptive nonlinear CF scheme is used for fusing accelerometer and gyro measurements, the nonlinear CF damping ratio is fixed to 0.707, and its natural frequency is adaptively determined by an exponential function, and the function is correlated with dynamic intensity.

The performance of the estimator in several typical drilling modes were tested on a DPRSS prototype, the results indicate that the dual-accelerometer combined with the adaptive nonlinear CF is an efficient Toolface estimator for drilling engineering. The estimator extracts the most reliable components from the gyro and accelerometers, and provides a general and effective signal processing algorithm for downhole data. Future work would involve using the Dynamic Toolface estimator performance test in an actual drilling process.

## Figures and Tables

**Table 1:** Characteristics of Sensors.

Parameters	Accelerometer	Gyro
Range	±8 g	±1200°/s
Sensitivity	1024 counts/g	0.83 mV/°/s
Bandwidth	400 Hz	40 Hz

**Table 2:** RMSE of Different Toolface Estimations in Stick-slip Test.

Sources	Dual-Accelerometer	Gyro	Proposed CF
RMSE (°)	18.022	11.6306	1.0069

**Table 3:** RMSE of Different Toolface Estimations in Multi Processes Test.

Sources	Dual-Accelerometer	Gyro	Proposed CF
RMSE (°)	61.032	5.872	3.017
